# Modelling of C-terminal tail of human STING and its interaction with tank-binding kinase 1

**DOI:** 10.3906/biy-2108-90

**Published:** 2021-11-04

**Authors:** Rahaf ATA OUDA AL-MASRI, Hajara AUDU-BIDA, Şebnem EŞSİZ

**Affiliations:** Kadir Has University, Faculty of Engineering and Natural Sciences, Department of Molecular Biology and Genetics, İstanbul, Turkey

**Keywords:** Loop modeling, cGAS-cGAMP-STING pathway, stimulator of interferon genes (STING), Tank-binding kinase 1 (TBK1), C-terminal tail (CTT) domain, protein-protein docking

## Abstract

Stimulator of interferon genes (STING) plays a significant role in a cell’s intracellular defense against pathogens or self-DNA by inducing inflammation or apoptosis through a pathway known as cGAS-cGAMP-STING. STING uses one of its domains, the C-terminal tail (CTT) to recruit the members of the pathway. However, the structure of this domain has not been solved experimentally. STING conformation is open and more flexible when inactive. When STING gets activated by cGAMP, its conformation changes to a closed state covered by 4 beta-sheets over the binding site. This conformational change leads to its binding to Tank-binding kinase 1 (TBK1). TBK1 then phosphorylates STING aiding its entry to the cell’s nucleus.

In this study, we focused on the loop modeling of the CTT domain in both the active and inactive STING conformations. After the modeling step, the active and inactive STING structures were docked to one of the cGAS-cGAMP-STING pathway members, TBK1, to observe the differences of binding modes. CTT loop stayed higher in the active structure, while all the best-scored models, active or inactive, ended up around the same position with respect to TBK1. However, when the STING poses are compared with the cryo-EM image of the complex structure, the models in the active structure chain B displayed closer results to the complex structure.

## 1. Introduction

Human Stimulator of Interferon Genes (STING) protein is a homodimer, 379 amino acid long transmembrane protein encoded by the human TMEM173 gene. It is expressed in hematopoietic cells and immune tissue (Barber, 2015). STING is a member of an immune response signaling network that gets activated in response to bacterial, protozoa, viral nucleic acids, and self-DNA through the regulation of type-I interferon (IFN) (Li, Wilson and Kiss-Toth, 2017)

**Figure 1:**
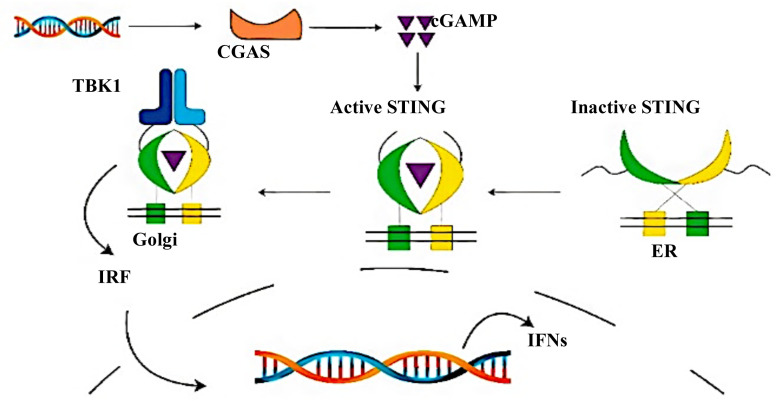
cGAS-cGAMP-STING pathway.

Structurally, STING is divided into three parts, an N-terminal domain that includes four transmembrane regions (1–154), which functions as a control for interorganelle trafficking and membrane anchorage, a dimerization and ligand-binding domain (155–342), and a C-terminal tail (CTT, 343–379) that is located in the cytoplasm. CTT domain includes the conserved PLPLRT/SD motif and pLxIS motif that STING uses to recruit other members of the pathway (Zhao et al., 2019). In a healthy human cell, DNA is located either in the nucleus or mitochondria, but, in some rare cases, the mtDNA/DNA is released to the cytosol. This triggers the cGAS-cGAMP-STING pathway which gets also activated by bacterial, protozoa, or viral nucleic acids double-stranded DNA (dsDNA). The pathway as explained in Figure 1, starts with the dsDNA binding to cyclic GMP-AMP synthase (cGAS) that uses ATP and GTP to catalyze the formation of cGAMP, a second messenger(Bai and Liu, 2019; Unterholzner and Dunphy, 2019). The catalyzed cGAMP binds to STING in the ER activating it.

**Figure 2:**
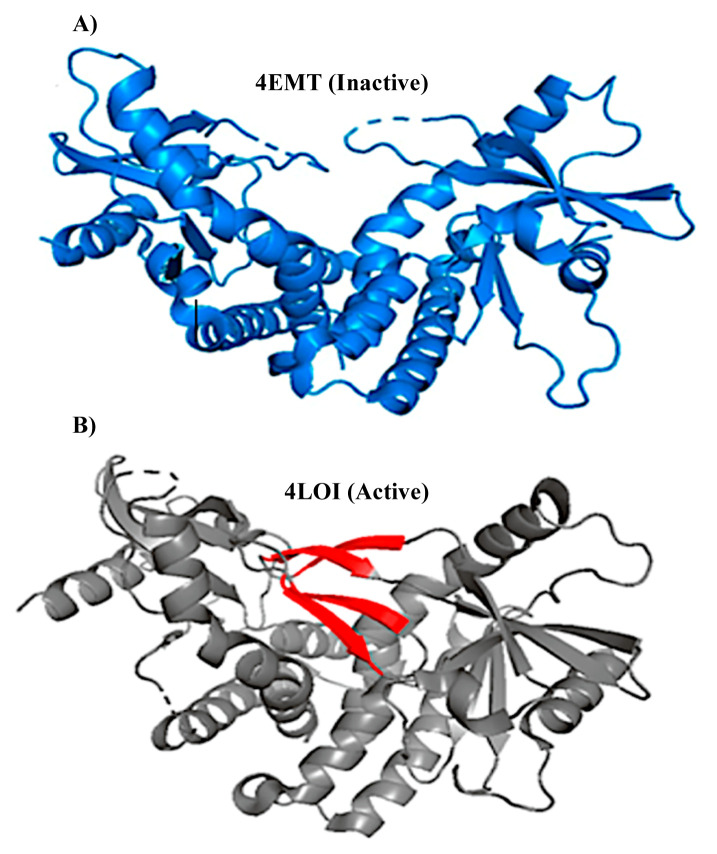
A) Inactive STING structure (4EMT). B) Active STING structure (4LOI) with the β-sheets coloured in red.

In Figure 2, inactive (Shu et al., 2012) STING conformation is displayed with the active X-ray structure (Gao et al., 2013). These structures are formed by dimerization of two monomers and ligand-binding domain (155–342). There is also a membrane-spanning segment and CTT domain, which are not resolved in these structures. STING is a homodimer, and when cGAMP binds to STING, the two monomers of the STING come closer around the ligand-binding site. This closure over ligand forms four β-sheets, by bringing 2 β-sheets from each subunit (four beta-sheets in red, Figure 2B). This conformational change leads to the CTT domain being released and moving towards the lid residues. Only then, STING will bind to TBK1 (Zhao et al., 2019). However, the CTT domain of STING is still not fully understood nor has its crystal structure been solved. This limits our understanding of STING-TBK1’s complex formation and its important functional interactions.

**Figure 3:**
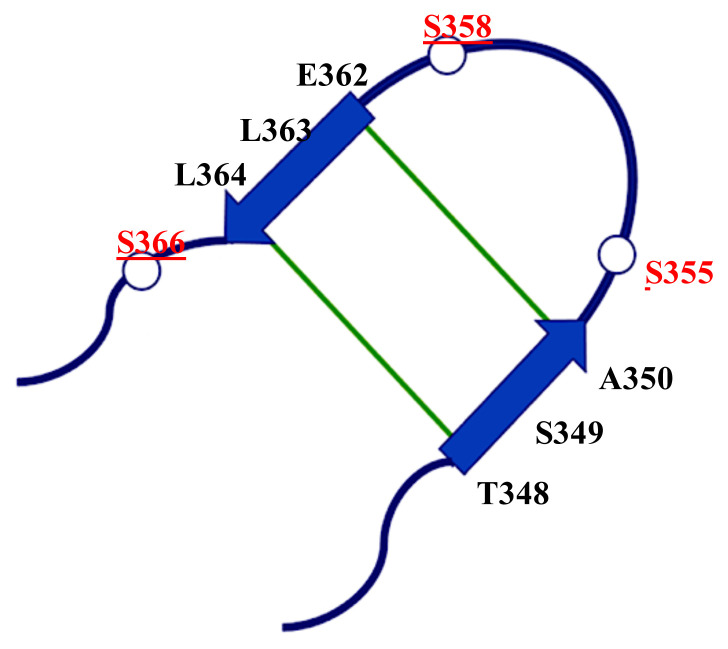
The CTT structure in the cGAMP-bound form at 780 ns (Tsuchiya et al., 2016). The hydrogen bonds were shown by green lines. The conserved SER residues in mouse and human STING are underlined.

In the molecular dynamics (MD) study of Tsuchiya and coworkers (Tsuchiya et al., 2016), active (cGAMP-bound) and inactive STING structures with modeled CTT loop structures were used. 700 ns and 1000 ns-long MD simulations in explicit solvent molecules were collected for each structure. In the cGAMP-bound structure, a more organized and structured CTT loop structure is observed. Mainly, a temporary β-sheet structure is formed between residues 348–350 and 362–364 as seen in Figure 3. Hydrogen bond interactions between Thr348-Leu364 and Ala350-Glu362 main chain are observed only in the active structure. Tsuchiya and coworkers proposed that the formation of this β-sheet might be an important factor in the complex formation of TBK1 and STING (Tsuchiya et al., 2016). However, they did not have TBK1 in these simulations; only STING structures were present.

**Figure 4:**
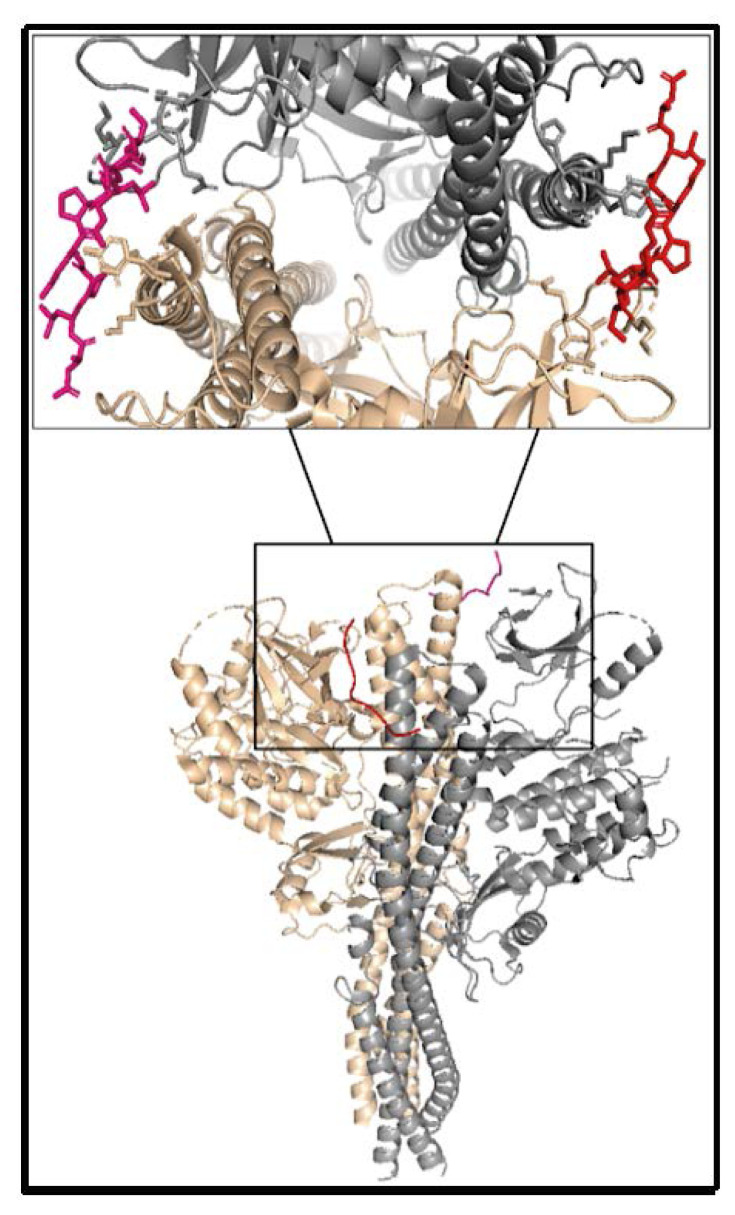
Crystal structure (PDB code: 6NT9) of the complex structure of STING-TBK1. STING chain A (red), STING chain B (pink), TBK1 chain A (wheat), and chain B (grey).

When the TBK1-STING complex structure is considered, a recent cryo-EM structure at 3.3 Å resolution was obtained in 2019 (Zhang et al., 2019). This complex structure is in between human TBK1 and chicken STING, and the resolution of the STING part is lower than 3.3Å. Thus, in the complex structure, PDB code 6NT9, only the STING tail is resolved while the whole TBK1 is in all-atom detail (as seen in Figure 4, red and pink for STING tails). The resolved segment of STING is only 8 amino acids long out of the 36 amino acid CTT loop. Namely, a high-resolution complex structure of human TBK1 and human STING structure is still missing.

In this study, we focused on modeling the CTT domain in both active and inactive STING conformations by homology and loop modeling. Then, we docked full-length STING with the CTT domain in multiple conformations to TBK1 by using HADDOCK software. We used the resolved parts of STING as restraints in the protein-protein docking part. Finally, we analyzed and compared the structural features of STING-TBK1 complex formation in the active and inactive forms of STING.

## 2. Method

### 2.1. Modeling

**Figure 5:**
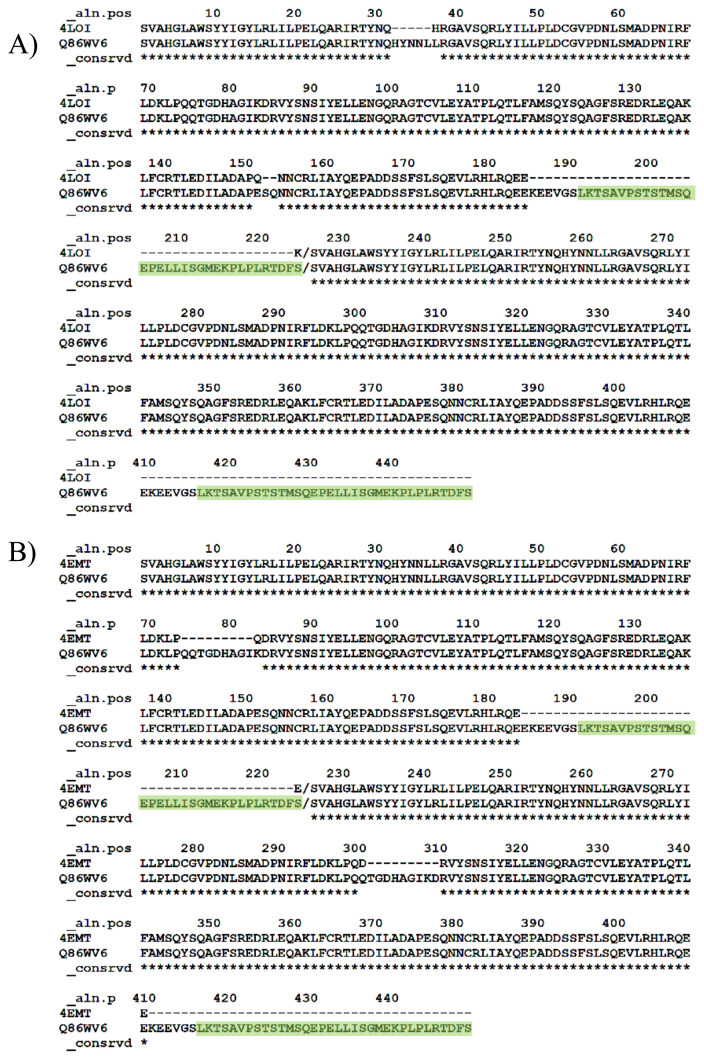
The alignment of (A) Active structure and target sequence. (B) Inactive structure and target sequence. Green highlighted sequences are the two CTT chains.

Active STING (PDB code: 4LOI) and inactive STING (PDB code: 4EMT) structures shown in Figure 2 were used as the templates to model STING structure with the CTT domain. These two structures are X-Ray structures for human STING, but they lack the CTT loop part. CTT domain is approx. 36 amino acid long loop, and it exists at the end of each monomer of the dimer structure. For building the homology models and for loop modeling, MODELLER auto-model and loop classes were used (Webb and Sali, 2016). First, each X-Ray structure was aligned with the full sequence of STING (UniprotKB: Q86WV6) containing the CTT domain. Figure 5 shows the alignment results, and the missing CTT sequence is highlighted in green in the PIR sequence format. We, then, built ten models for each template by using the alignments. Next, the best model was picked based on the Z-Dope score. Z-Dope is a statistical score developed by MODELLER, which evaluates the energy of the models through many iterations. Models returning the minimum value of normalized Z-Dope (−1.0 being the native-like structure) score were chosen as the most probable structure.

Subsequently, two best models, one for active and one for inactive, are carried to the loop modeling step. Here, many random loop structures were generated by randomizing the atomic positions by ±5Å in each Cartesian direction. In the loop modeling algorithm, the model optimization occurs twice; the first takes into consideration only the loop atoms, while the second iteration takes into consideration how the atom interacts with the rest of the protein. For each structure, 50 different loop conformations were built via the MODELLER loop modeling module used (Fiser, Do and Šali, 2000).

MODELLER has different levels of MD refinement stage after the optimization of the models. This is separate from the initial model building step. The models obtained after the loop modeling step was refined by setting “md_ level” parameter to “very_slow” in MODELLER. This is equivalent to 10000 steps of minimization followed by 1 ns equilibration of the model at 300 K. Again, for these structures, only the loop parts are different.

After the modeling step, loop modelling, and refinement stages, we picked the best scored inactive and active STING structure to protein-protein docking step. The details of filtering the best structures are explained in the results section.

### 2.2. Protein-protein docking

With the information we have about STING-TBK1 interaction, the protein-protein docking step was done using HADDOCK (Dominguez, Boelens and Bonvin, 2003). To drive the docking process, HADDOCK introduces ambiguous interaction restraints (AIRs) which represent the distances between the residues involved in the interaction of two proteins. In our case, these interacting residues were taken from PDB structure of 6NT9 (Zhang et al., 2019). Mainly, HADDOCK algorithm introduces three different steps: Randomization of orientations and rigid-body minimization followed by semiflexible simulated annealing in torsion angle space. In the first stage of minimization, AIRs are included in the energy function being minimized. The following scoring function is utilized in the first step:


$$SCORE=E_{vdw}+E_{elec}+E_{AIR}$$

In this equation, van der Waals energy, electrostatic energy, and the distance restraint contribution of AIRs are included. The best structures from this step were taken to torsion angle space. In the final refinement stage, the following scoring function is evaluated for the complex structure.


$$SCORE=1.0E_{vdw}+0.2E_{elec}0.1E_{dist}+1.0E_{solv}$$

Finally, a refinement step in cartesian coordinate space with explicit solvent (TIP3P water model) is performed.

For docking, the STING segment was removed from the cryo-EM complex structure. The remaining structure was used for TBK1, and it has a resolution of 3.3 Å. For STING the models we built with the CTT domain in active and inactive conformations were used.

The active residue numbers directly involved in the binding were taken from the cryo-EM complex and set as (residues 215, 217, 218, 220, 219) for STING and (residues 8, 27, 29, 577, 581, 584, 582) for TBK1. The remaining passive residues were set automatically by HADDOCK. For the docking, all HADDOCK parameters were left as default. HADDOCK yielded seven cluster results. The cluster with the lowest z-score was considered as the best structure. We also analyzed the results by superimposing them onto 6NT9 to see how close our STING active residue poses are to the crystal structure’s active residues. Moreover, STING has two chains to be docked to TBK1, chain A, and chain B, and the docking process was repeated several times using different chains.

Additionally, the best poses obtained from HADDOCK are cross-checked with the ClusPro web server (Kozakov et al., 2017). ClusPro uses rigid-body protein-protein docking, while HADDOCK incorporates rigid and flexible steps into the docking. Still, for the validation of the poses, a second protein-protein docking software was needed. Restraints in HADDOCK were given to the ClusPro web server as attraction points on the surfaces of TBK1 and STING. The obtained poses are then compared with the superposed HADDOCK results.

## 3. Results

### 3.1. Modelling

**Figure 6:**
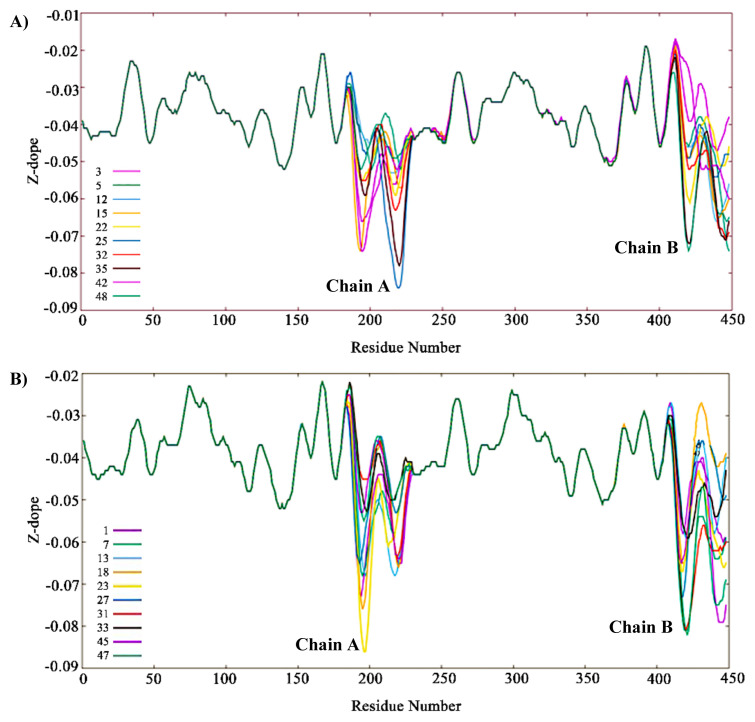
Z-Dope profiles of the top 10 models. A) Active structure. B) Inactive structure. Top 10 models are displayed with 10 different colours.

**Figure 7:**
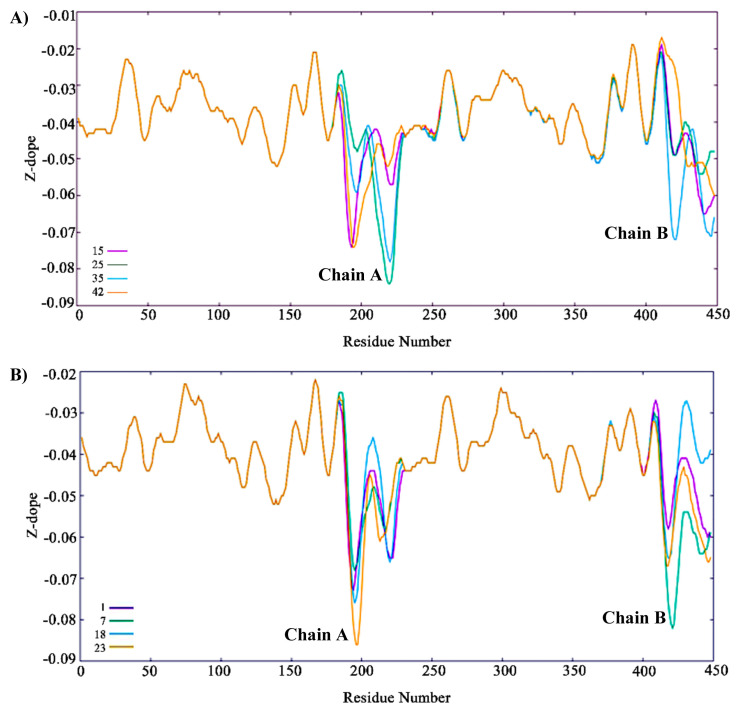
Z-Dope profiles of the top 4 models. A) Active structure. B) Inactive structure. Top 4 models are displayed with 4 different colours.

**Figure 8:**
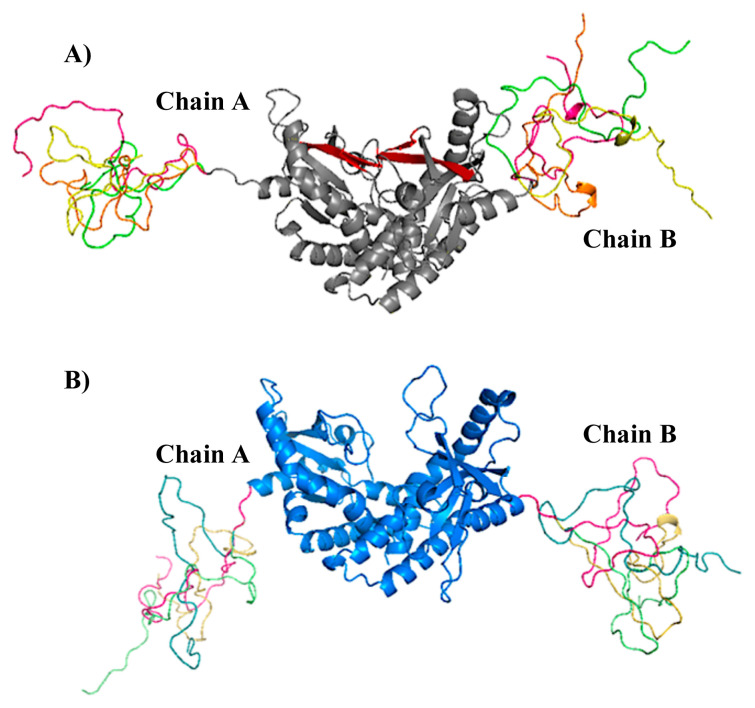
STING models with the modeled CTT domain. A) Active conformation (beta sheets are displayed in red). B) Inactive conformation.

At the end of the loop modelling step, we had 50 different conformations for each loop. In Figure 6A, the highest Z-Dope scored loop structures are displayed for chain A and chain B in the active structure. In Figure 6B, the same type of analysis is displayed for the inactive structure. Z-Dope score changes between −1 and 1, and a lower score means native-like structure. In these graphs, Z-Dope profiles, smoothed over a 15-residue window, and normalized by the number of restraints acting on each residue, are displayed. This shows the local quality of the model around each residue. We picked the best 4 structures from Figure 6 for both loops to be evaluated in the next step (Figure 7). A ribbon representation of the top 4 models superimposed in Figure 8 shows the difference in the loops for both the active and inactive models. Please note the loops are staying lower with respect to the body of the protein in the inactive structure (Figure 8A is for active, and Figure 8B is for inactive). This conformational difference has been determined to be functionally important for STING to interact with TBK1, STING is moving its arms up (its CTT loops) when it is activated and associating with TBK1 (cf. Figure 1, cGAS-cGAMP-STING pathway).

**Figure 9:**
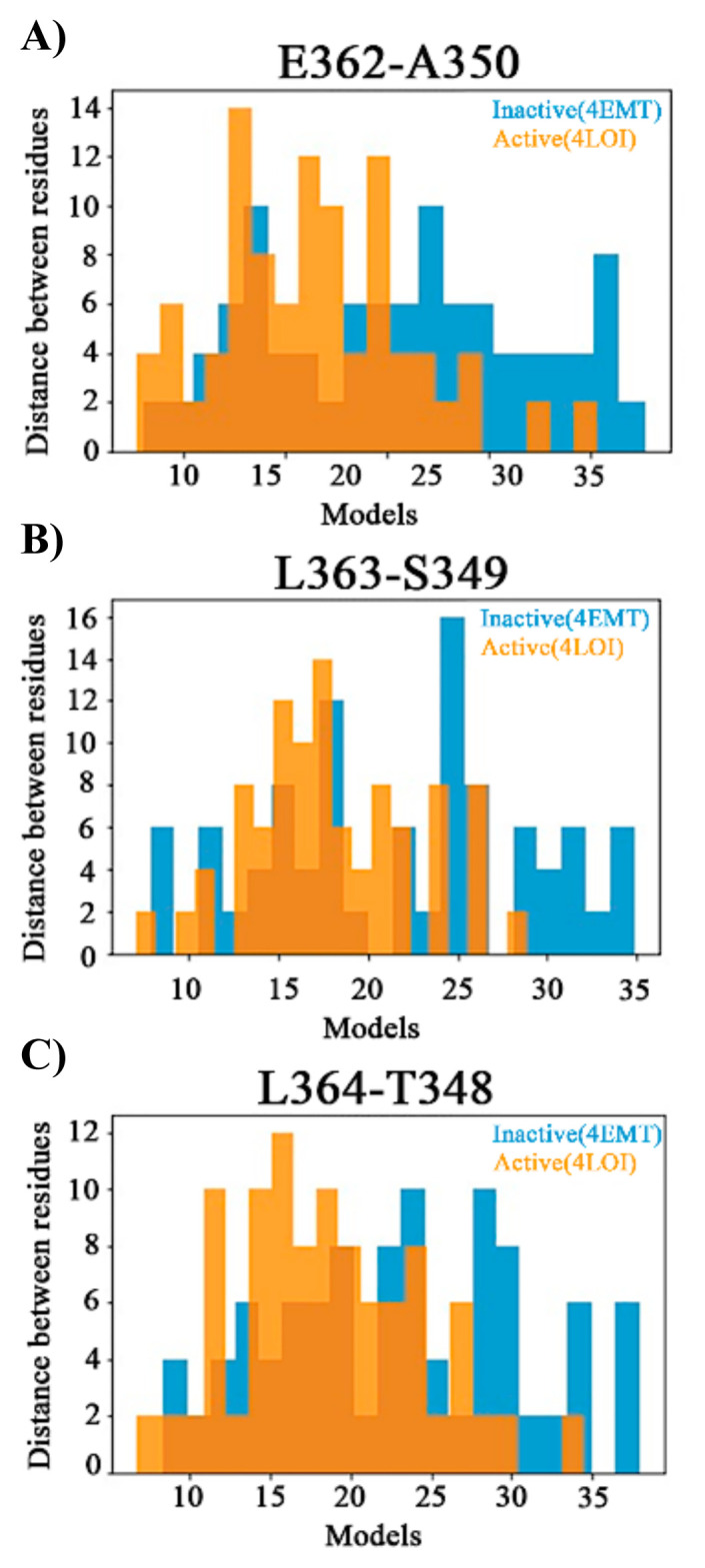
β-sheet forming pairs. Distance distribution of A-E, S-L, and T-L residues respectively in 50 models. Inactive (blue), active (orange).

Also, the formation of ordered structures in the loop region is inspected. In a previous MD study, a partial β-sheet formation in the CTT region has been observed only in the active structure of STING (Tsuchiya et al., 2016). Figure 9 displays distance distributions of the residues in the small β-sheet segment active (orange) and inactive (blue). The distances are between E362-A350, L363-S349, and L364-T348 (shown in Figure 3). Figure 9A displays the distance distribution of E362-A350 in all loops, and the distances are shorter in the active structure. Figure 9B displays L363-S349 distance, and a more random distribution is observed in both active and inactive structures. The inactive structure still samples larger distances. And finally, in Figure 9C, L364-T348 distance distribution is displayed, and in the active structure, this distance is also located more to the left. As a summary in the active structure, the previously observed β-sheet segment stays more compact. Please note that these loops are very mobile and random, and the sheet formation in the MD study is not checked experimentally. But still, our study also suggests that the distances in this specific region stay closer in the active structure.

**Figure 10:**
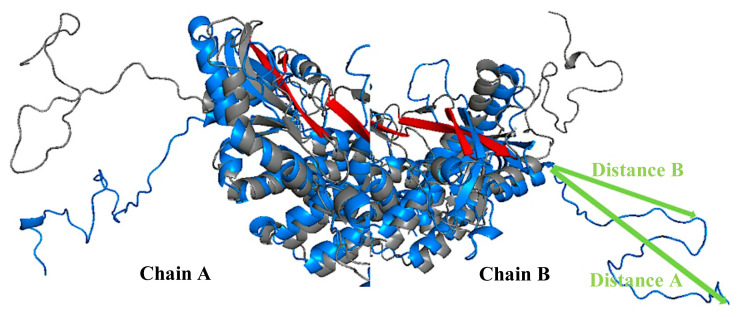
β-sheet best models. A) Best models for chain A. B) Best models for chain B. Green arrows show half distance and full distance of CTT loop. Active (grey), inactive (blue).

Additionally, the best inactive and active models according to this β-sheet distances were aligned and can be seen in Figure 10. These models are the ones that displayed the shortest distance for β-sheet forming residues. The loops are staying more compact in the active structure (grey colored in Figure 10) while the arms as CTT loops are again lower in the best inactive model (blue colored in Figure 10) structure when compared with the active structure.

By considering all the above-mentioned criteria for the structural properties of the CTT loop, Z-Dope score, CTT loop height, and β-sheet distances are combined in Table 1. Z-Dope scores plotted in Figure 6 and Figure 7 were local scores. They showed the quality of local regions. However, the values in the Table 1 are the overall scores of the complete loop sections. CTT height is measured from the end of the CTT loop to the beginning of the loop (distance A), and from the midpoint of CTT to the beginning of the loop (distance B). In Figure 10B, these two distances are shown with green arrows in one of the models.

For inactive STING models chain-A, model 46, and model 11 have the shortest distances in β-sheet for chain A. Those two models have also the most compact form in CTT-height and low Z-Dope scores. The other model, model 23, has the lo west Z-Dope scores as well as compact CTT height. That means the most compact loop structure is giving the best Z-Dope score also.

For inactive STING models, chain B, model 5, and 19 have the shortest distances in β-sheet and relatively short CTT height. The best Z-Dope score with −0.566 has also a short CTT height. From the analysis of these two sets of models, the best Z-Dope scores are also resulting in low CTT heights.

For active models, Z-Dope scores are much better than inactive models in general. Once again, the TBK1 binds to STING from the CTT loop in the active conformation. Here in the results of active models, the loop structures are organized and more compact according to Z-Dope scores. In chain A model 12 has a strikingly low distance for β-sheet formation as well as the lowest Z-Dope score. For that model, the CTT model height from the midpoint (distance B in the table) distance is also relatively low. For chain B, the same model, model 12 is giving good results. Additionally, model 9 is giving short distances in β-sheet formation, as well as low CTT height values for chain B.

### 3.2. Protein-protein docking

Since Z-Dope scores for different models are still very close to each other; for the protein-protein docking steps, instead of taking single best models, we took all the models in Table 1 to HADDOCK analysis. Models in Table 1 were taken to HADDOCK database for the docking of STING and TBK1. The STING chains A and B were docked separately to TBK1 binding site.

In Table 2, the HADDOCK results are displayed with HADDOCK scores for the best clusters obtained and the corresponding Z-score of each cluster. RMSD values are calculated after superposing the residues of the whole TBK1 and STING segment that are solved in cryo-EM complex structure (PDB code:6NT9).

**Figure 11:**
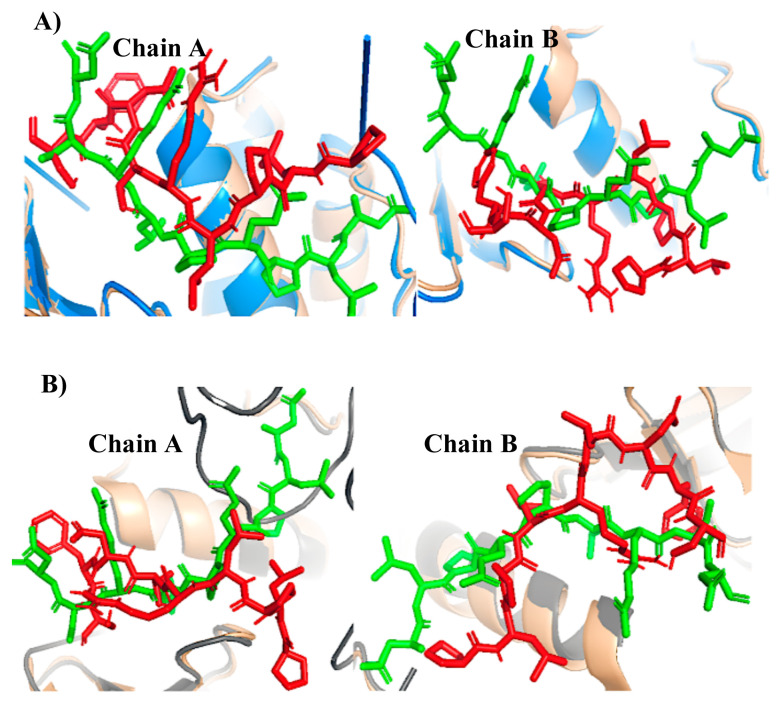
Docking poses of the STING chains (in red) and PDB complex cryo-EM segment (in green). A) Inactive. B) Active.

Model 46 chain A and Model 27 chain B for the inactive conformation and Model 12 chain A, Model 35 chain B for the active conformation displayed the best RMSD results from the docking step. Model 12 for the active model is also the best model according to the Z-Dope score. In Figure 11A and B, the poses of the STING segments are displayed for inactive structure and active structure, respectively. The RMSD values are calculated superposing TBK1 and cryo-EM resolved STING segment (green colored in the figures). Since the STING cryo-EM structure is for chicken, and our models are for human, the RMSD values are for different sequences but include the conserved PLPLRT segment of CTT loops.

**Figure 12:**
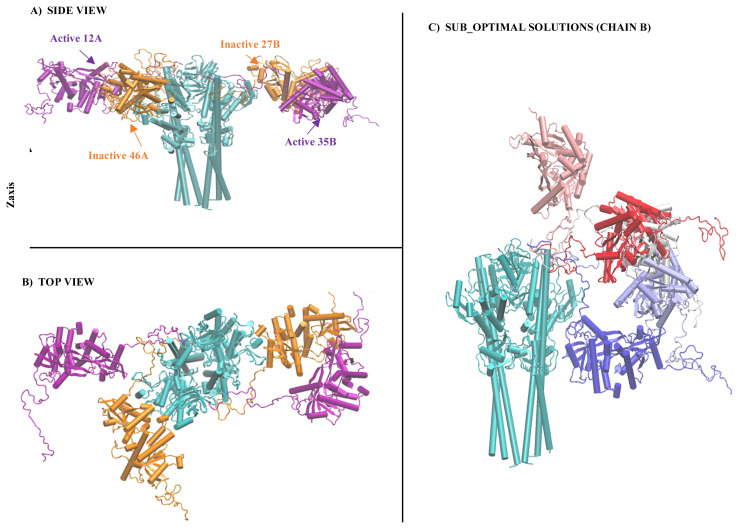
The HADDOCK models with the lowest RMSD. The inactive models are represented in purple, while the active models are in orange. TBK1 is in cyan. A) Side View. B) Top View. C) Not best but some of the suboptimal docking poses of STING with different colouring.

The best models according to RMSD values are shown in Figure 12 as full-length complexes to display the location of STING with respect to TBK1. TBK1 is cyan, while active STING molecules are purple and inactive ones are orange in this Figure. One important finding is that all those models are docked approximately around the same position in terms of the z-axis of TBK1. Please don’t forget that there is a long CTT loop; thus, the STING molecule has all the freedom to be docked. In other words, out of 36 residues modelled in the CTT loop, we are only giving the 8 residue segments as restraints to the HADDOCK. In Figure 12C, different poses that we obtained for STING as sub-optimal solutions in HADDOCK for chain B are displayed (TBK1 is cyan, and different poses of STING are displayed with colours ranging from white-red-blue scale in Figure 12 C). Out of all those possible locations, both inactive and active best-docked poses ended up in the same location with respect to TBK1(Figure 12 A)

**Figure 13:**
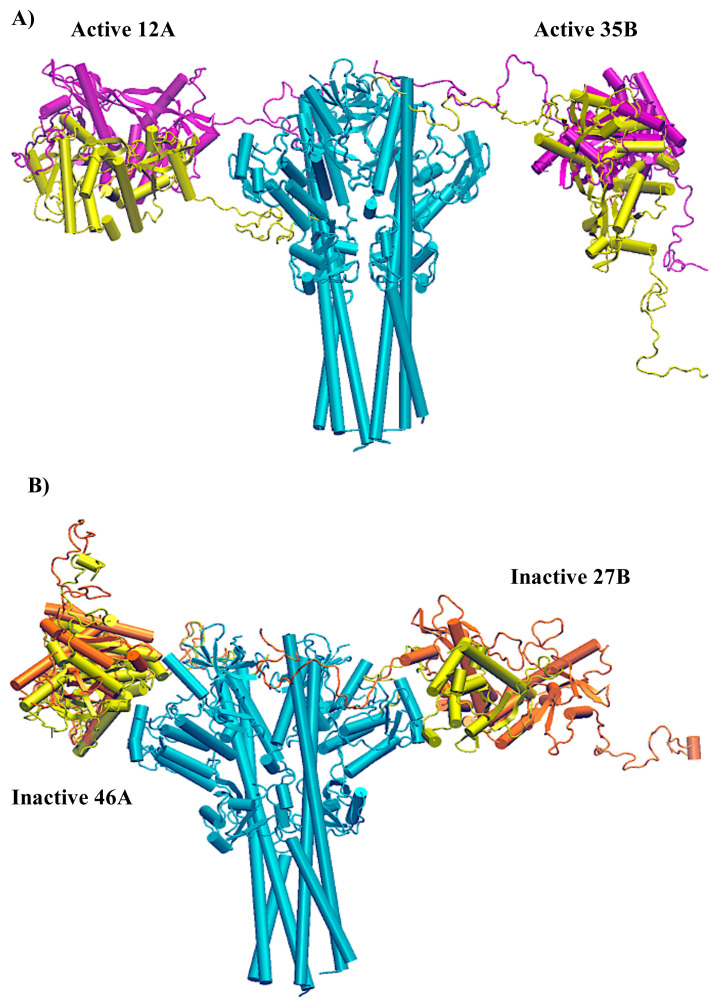
ClusPro poses together with HADDOCK poses. A) Active models (HADDOCK in purple, ClusPro in yellow). B) Inactive models (HADDOCK in orange, ClusPro in yellow).

We also checked our findings here with a second protein-protein docking software, ClusPro. Figure 13 displays the best-scored models in HADDOCK together with the results of ClusPro. We took the best-scored STING models and one TBK1 structure and re-dock them in ClusPro. We used the restraints from HADDOCK as attracting residues in ClusPro and left all the other parameters as default. The best or at most second best pose in ClusPro gave a similar position of STING with respect to TBK1 (Yellow colored STING in Figure 13 is ClusPro results).

Another key finding of this study is that in all the active models, when STING is docked from Chain B (see Table 2 Active STING chain B), RMSD values are lower than 8 Å. Thus, the active models, when the structure of STING is more compact, have better poses. We didn’t get similar results for the inactive structure or active structure but chain A. There is still an ongoing discussion whether STING is binding to TBK1 from two CTT chains or one. If one STING molecule is interacting with TBK1 from two sites, then the second CTT loop can have different behaviour when the first chain is bound (Zhang et al., 2019; Zhao et al., 2019).

## 4. Discussion

When the STING molecule binds to cGAMP, it gets activated. Then, it can interact with TBK1. However, the interaction mechanism of STING with TBK1 remains unclear. In this study, we modelled the missing CTT loop in the human structure, which is around 36 amino-acid long by using loop modelling algorithms in MODELLER software. In general, loops are very difficult to model or resolve by experimental techniques due to their random and highly flexible structures (Fiser, Do and Šali, 2000; Lee, Heo and Seok, 2016; Feig, 2017). Here, instead of obtaining an initial homology model for the loop segment, then sampling it with different simulation techniques, we obtained an ensemble of loop structures from MODELLER program directly. Then, they are analysed with different scoring functions. These different loop structures could have also been obtained from a simulation such as MD simulation; however, MODELLER loops are sampling a larger pool of geometries with lower precision. Thus, in this study, we aimed at a larger sample of conformations that might be less precise in the atomistic details.

The loop structures are first analysed with the MODELLER scoring function locally and globally. Z-Dope scores obtained for active models are much lower than inactive models in general. Additionally, the height of the CTT and secondary structure formation of a small β-sheet, previously observed in 700 ns molecular dynamics simulation, has been checked for the models. The ligand-induced ordering of the CTT was observed only in the active conformation (Tsuchiya et al., 2016). In our models, the distance required to form this ordered structure is checked via the distribution of three distances in the models and we also observed that in the active conformation, these distances stayed shorter. Finally, the height of CTT, like the arms of the molecule, is observed to be higher in the active conformation.

Last but not least, in the HADDOCK protein-protein docking step, all the best-docked results ended up around the same vicinity of TBK1 (Figure 12 A). The location of TBK1 with respect to STING is still not known. Only a small conserved region of chicken STING is solved in the cryo-EM image (Zhang et al., 2019; Zhao et al., 2019). In our findings, RMSD values of the active human STING conformations for chain B are closest to the cryo-EM image structure. In the inactive models, there are also similar poses however, RMSD values are not that close.

As a summary, STING’s relative location to TBK1 did not change whether STING is the inactive or the active form. However, the CTT tail sampled much closer results to cryo-EM complex structure when STING is in active conformation. We hope the findings of this study, especially the location of STING with respect to TBK1 and binding of one of the chains of CTT to TBK1 more efficiently than the second chain, will be tested when the full-length STING-TBK1 complex structure becomes available. Moreover, when creating the model in MODELLER and docking in HADDOCK, the solvation effects are implicitly included in the scoring functions. In a future study, we are planning to run the best scored complexes of this study in a fully solvated all-atom molecular dynamics simulation to observe the effect of solvation and equilibration on the CTT loop structure.

## Figures and Tables

**Table 1:** The models with the lowest Z-Dope scores, distance between the temporary β-sheet residues and CTT height are displayed in the table.

Models		Distance between temporary β-sheet residues (chain A)			Distance between temporary β-sheet residues (chain B)			Z-Dope score	CTT-Height	
		A-E	S-L	T-L	A-E	S-L	T-L		Point A	Point B
Inactive STING (chain A)	46	7.97	8.41	8.41	15.73	11.13	14.04	−0.54	64.38(A)	41.46(A)
	36	23.81	24.79	27.87	27.06	25.52	23.80	−0.48	89.21(A)	46.30(A)
	11	11.78	8.83	9.95	19.11	24.85	27.64	−0.55	68.88(A)	53.16(A)
	23	27.08	29.32	33.48	25.16	24.79	28.32	−0.56	45.72(A)	42.58(A)
	25	29.17	31.89	37.89	19.97	23.27	25.00	−0.52	54.82(A)	64.74(A)
Inactive STING (chain B)	19	22.54	22.26	21.62	9.28	9.20	11.53	−0.51	45.61(B)	62.99(B)
	27	20.34	18.12	22.66	22.41	24.31	29.98	−0.53	49.63(B)	24.47(B)
	33	12.02	7.82	12.13	18.93	19.30	23.77	−0.57	42.77(B)	32.99(B)
	5	18.39	18.57	21.98	11.48	12.20	10.03	−0.52	65.65(B)	30.49(B)
	7	25.18	29.84	29.75	34.28	37.03	42.82	−0.51	51.26(B)	16.59(B)
Active STING (chain A)	11	8.99	12.62	8.25	14.99	19.18	17.69	−0.78	44.95(A)	53.16(A)
	36	8.11	13.88	6.75	24.03	26.80	28.00	−0.74	56.24(A)	47.75(A)
	12	12.84	12.53	12.00	12.84	11.64	14.83	−0.81	56.96(A)	36.46(A)
	26	7.68	13.87	15.26	8.93	12.87	10.59	−0.79	65.36(A)	40.57(A)
	25	17.43	26.56	16.49	18.42	21.76	27.25	−0.77	79.86(A)	34.54(A)
Active STING (chain B)	12	12.84	12.53	12.00	10.37	11.64	14.83	−0.81	63.56(B)	41.59(B)
	38	19.38	17.60	18.34	28.33	30.33	35.99	−0.79	71.96(B)	18.21(B)
	1	12.92	15.36	15.09	14.02	19.47	18.14	−0.80	52.37(B)	32.89(B)
	9	16.62	17.77	16.16	10.83	7.65	12.66	−0.77	32.74(B)	23.20(B)
	35	15.95	21.34	19.16	27.18	26.14	26.34	−0.73	67.97(B)	31.67(B)

**Table 2:** The models with the lowest HADDOCK scores and Cluster Z-Scores with their RMSD values are displayed in the table below.

Models		Cluster Z-Score	RMSD values	HADDOCK score	Models		Cluster Z-Score	RMSD values	HADDOCK score
Inactive STING (chain A)	46	−2.0	6.3	−105.8 +/− 7.0	Active STING (chain A)	11	−1.4	14.5	−80.5 +/− 2.1
	36	−1.8	10.6	−94.0 +/− 2.1		36	−2.1	17.5	−96.7 +/− 4.5
	11	−1.5	16.8	−93.5 +/− 1.8		12	−1.6	8.0	−79.2 +/− 2.3
	23	−2.3	12.3	−79.0 +/− 1.9		26	0.0	10.2	−75.7 +/− 5.1
	25	−1.8	16.1	−75.0 +/− 2.7		25	−1.6	15.9	−100.6 +/− 0.3
Inactive STING (chain B)	19	−1.4	14.6	−70.8 +/− 1.7	Active STING (chain B)	12	−1.0	7.4	−65.4 +/− 0.6
	27	−1.7	6.7	−87.0 +/− 8.7		38	−1.4	6.8	−74.7 +/− 7.1
	33	−1.3	14.4	−78.9 +/− 1.8		1	−1.7	6.3	−77.9 +/− 7.2
	5	−1.7	8.5	−60.3 +/− 0.8		9	−2.1	7.3	−89.6+/− 1.7
	7	−1.6	11.9	−85.7 +/− 3.1		35	−1.5	5.3	−74.7 +/− 2.1
